# Clinical Holistic Medicine: Tools for a Medical Science Based on Consciousness

**DOI:** 10.1100/tsw.2004.34

**Published:** 2004-05-26

**Authors:** Søren Ventegodt, Mohammed Morad, Niels Jorgen Andersen, Joav Merrick

**Affiliations:** ^1^ The Quality of Life Research Center, Teglgårdstræde 4-8, DK-1452 Copenhagen K, Denmark; ^2^ Division of Community Health, Ben Gurion University, Beer-Sheva, Israel; ^3^ Norwegian School of Management, Sandvika, Norway; ^4^ National Institute of Child Health and Human Development, Office of the Medical Director, Division for Mental Retardation, Ministry of Social Affairs, Jerusalem and Zusman Child Development Center, Division of Pediatrics and Community Health, Ben Gurion University, Beer-Sheva, Israel

**Keywords:** quality of life, QOL, philosophy, human development, holistic medicine, public health, holistic health, holistic process theory, life mission theory, Denmark, Israel

## Abstract

Biomedicine focuses on the biochemistry of the body, while consciousness-based medicine — holistic medicine — focuses on the individual's experiences and conscious whole (Greek: holos, whole). Biomedicine perceives diseases as mechanical errors at the micro level, while consciousness-based medicine perceives diseases as disturbances in attitudes, perceptions, and experiences at the macro level — in the organism as a whole. Thus, consciousness-based medicine is based on the whole individual, while biomedicine is based on its smallest parts, the molecules. These two completely different points of departure make the two forms of medicine very different; they represent two different mind sets and two different frames of reference or medical paradigms. This paper explains the basic tools of clinical holistic medicine based on the life mission theory and holistic process theory, with examples of holistic healing from the holistic medical clinic.

## INTRODUCTION

This paper is the practical pendent to a series of theoretical papers on quality of life as medicine and holistic medicine to be reviewed below. After the short review, we explain the three basic tools of consciousness-based medicine, where feelings are at the core of holistic medicine. Holistic medicine is dealing with man as a whole and this wholeness, global level of existence, or “soul” is integrating all the being, having, and doing of the individual. We believe this top level of the biological organism to be the seat of consciousness, which is the reason why we focus on consciousness in our holistic medicine. The first step for the patient is therefore to reach inside the body to reveal the feelings and impressions hidden there. They are often rooted in the personal history (unfinished issues or the so-called gestalts). The next step is to verbalize the feelings, to understand, and finally let go of the life-limiting perspective in order to find a new perspective, which is more nourishing and supporting for life. We will see how this is done in a case story. Based on this example, we will analyze the main differences between biomedicine and consciousness-based medicine.

As is demonstrated in other articles in our series on clinical holistic medicine, there are still a number of important aspects of being a person and working on one’s health that are difficult to fit into holistic theories, so the job of developing the new medicine is far from finished. We hope that the medical community will engage in the exciting challenge of making consciousness-based medicine work in the medical clinic, hopefully to the benefit of thousands of chronic patients not helped sufficiently by biomedicine or alternative treatment as we know it today.

It is important to stress that the word holistic has been given many different meanings, so there are many different kinds of “holistic medicine”. Often spirituality has been stressed in holistic medicine, but the meaning of this word has not always been clear. In our version of holistic medicine, spirituality is *the abstract.* The life mission theory[1,2,3,4,5,6] states that the essence of man is his life purpose and this is the abstract core of being. The ideal contact between two persons is the contact we call “love”, so important in our clinical work, where our wholeness (“soul") openly and in full acknowledgment meets the other person’s wholeness (“soul”). This is a meeting “soul to soul”, where we connect as deeply as humanly possible. In a way, we merge our consciousness with the consciousness of the other, with the intention to serve this other person while realizing our own purpose of life, which is always about creating value for the other[2].

## THE BASIS FOR CLINICAL HOLISTIC MEDICINE

The life mission theory[1,2,3,4,5,6] states that everybody has a purpose of life, or a huge talent. Happiness comes from living this purpose and succeeding in expressing the core talent in your life. To do this, it is important to develop as a person into what is known as the natural condition, a condition where you know yourself and use all your efforts to achieve what is most important for you. The holistic process theory of healing[7,8,9,10] and the related quality of life theories[11,12,13] state that the return to the natural state of being is possible, whenever the person gets the resources needed for existential healing. The resources needed are, according to these theories, holding in the dimensions: awareness, respect, care, acknowledgment, and acceptance with support and processing in the dimensions: feeling, understanding, and letting go of negative attitudes and beliefs. The preconditions for holistic healing to take place are trust and the intention that the healing will take place. Existential healing is not a local healing of any tissue, but a healing of the wholeness of the person, making him much more resourceful, loving, and knowledgeable of himself, his own needs and wishes. In letting go of negative attitudes and beliefs, the person returns to a more responsible existential position and an improved quality of life. The philosophical change of the person healing is often a change towards preferring difficult problems and challenges, instead of avoiding difficulties in life[14,15,16,17,18,19,20,21]. The person who becomes happier and more resourceful often also becomes more healthy, more talented, and more able to function[22,23,24].

## TWO SIMPLE CLINICAL EXAMPLES

> **Female, aged 35 years – Feel!** The patient presents with neck pain and tensions in the thoracic and intercostal muscles [the muscles between the ribs]. We talk about the presence of a feeling, which the patient does not want to acknowledge. EXERCISE: Sit down for 10 min every day with your eyes closed and sense your feelings. Make room for all negative and positive feelings.

Another physician might refer this woman to a physiotherapist and prescribe analgesics if the neck pain is severe. Contrary to this, we recommend that she take the time to listen to her emotional life. Unprocessed feelings (anger, anxiety, or frustration) often manifest themselves as tensions in the body. If that is the case for this woman, we may be able to solve her troubling and probably recurring problems without any physical or chemical intervention.

That is how consciousness-oriented medicine differs from biomedicine. The biomedical physician typically resorts to medication and physical manipulation, while consciousness-based medicine will often begin by turning the patient's attention to the underlying emotional problems. Our feelings are a great source of knowledge of the unique causes of our diseases and health.

In modern society, we often fail to recognize the importance of feelings. We rely on reason and suppress many of the painful reactions to our less than perfect reality, which are basically natural and require some space. But suppressed feelings tie into knots and frequently we will not get rid of a symptom until we open ourselves to the repressed emotional pain.

> **Female, aged 20 years – abortion trauma.** Quality-of-life conversation. The patient presents with fatigue and persistent low spirits. "I feel bad about my body and myself" — low self-esteem — e.g., the statement "I am worthless". Had an abortion 6 months ago, which still troubles her. Cries on the couch, I hold her hand and she talks about the abortion. Conclusion: She has no close, intimate friends whom she trusts. A consequence of low self-esteem, which makes her certain that once she is seen, she will be rejected. Early problem when the patient felt rejected. EXERCISE: List of problems — describe your social, psychological, physical, educational, and sexual problems. EXERCISE: Write a description of the course of the abortion from beginning to end, and bring it back for follow-up conversation. PLAN: Feel, acknowledge, let go — for the next 6 months. Another appointment in 2 weeks.

It is estimated that there are at least 26 million legally terminated pregnancies each year throughout the world, at least 20 million illegally terminated with the result of at least 78,000 maternal deaths[25]. Every abortion is an emotional trauma that often is not processed and will torment the woman for years. This is a great shame, since the problem can be solved by relatively simple means. In this specific case, the problem was low self-esteem, a feeling with which so many young people struggle today. When we take a look behind the facade and view her self-esteem as the essential problem, she also will be able to see that her entire existence reflects her low self-esteem. The abortion is like an emotional plug in her and once it is removed, it releases a number of emotional problems that concerns her relationship with the significant individuals in her life.

As homework, the physician asks her to describe the abortion and make a list of all the problems in her life. These two exercises deal with the past and the present and, if successful, she will feel as if she has been relieved of a great burden. Once she has confronted the painful feelings, she will get a much clearer view of her position in life, and she will be able to let go of many negative attitudes towards herself and others.

In our view, these three steps are required to sort things out and heal: first, one has to *feel* the old emotional pain again, then clearly *acknowledge* the nature of the problem and where life took the wrong track, and finally *let go* of the negative attitudes to life that accumulate in all of us, when life is hard on us or we fail at something we want.

## HOLISTIC PROCESS THEORY[9]

We formulated these three steps — feel, acknowledge, and let go — following studies of therapeutic approaches of alternative therapists. What do alternative therapists do for their patients and clients? Well, either they touch the patients and help them feel the emotions and "energies" that are restrained in their bodies (for example, by means of massage, zone therapy, Rosen sessions, kinesiology, acupuncture, craniosacral therapy, bioenergy, primal therapy, or holotropic breathwork) or they help their patients verbalize their feelings and sensations and support them in acknowledging the structure of their lives (for example, through Gestalt therapy and other psychotherapy, cognitive therapy, transaction analysis, or existential group therapy sessions). They can also work with the patients' thinking and consciousness and help them towards new perspectives and life philosophies (for example, through Body Mirror System Healing, psychosynthesis, NLP, philosophical counselling, existential therapy, thought field therapy, and life philosophy courses such as our own summer courses "Life philosophy that heals" in Denmark). With these measures and techniques, alternative therapists can help the patients let go of physical tension, emotional tension, or tension in their minds.

We call the combination of these three essential steps: "feel, acknowledge, let go" holistic process theory. "Holistic" because it draws on the whole formed by the body, emotional life, and mind, and "process" because it describes the process where the pain load that a person has repressed earlier in life (from the spiritual to the physical level through life) once more becomes conscious and integrated. Thus, through the holistic healing process, one becomes aware of the causes of one's diseases and disorders, and at the same time as the misfortunes of the past are sorted out, one's quality of life, health, and functional capacity improve.

Let us provide an example from the clinic: a woman suffers from urinary tract infections that keep recurring and she is treated over and over again. (We see that in the clinic occasionally.) What could be the cause, seen from a holistic angle, and how should it be treated holistically?

As children we need love; without love we become anxious and insecure. If the anxiety becomes unbearable, we can escape from it by a "decision" that we are not worth loving. The pain may, in this perspective, be wrapped up and placed, for instance, in the skeletal muscles between the ribs or in the smooth muscles in the pelvis. Indeed, therapy often reveals that as adults, we carry the anxiety from our childhood hidden in the organs of the pelvis — in the intestine, bladder, sexual organs, or skeletal muscles.

If the anxiety is hidden away in the pelvis, it may weaken our bladder region and cause repeated or chronic infection of the urinary tract. To get rid of such chronic infection, the patient has to "become present in the pelvis" and feel the anxiety again. The gestalt — "the frozen now" with the original emotional pain — must be caught, verbalized, and made conscious. Finally, the patient must let go of his or her old perception of being unlovable. Not only will the cystitis go away as a result of this process, the patient will also attain higher self-esteem and thereby become easier to love. The patient has learned from his or her disease, the disease has been cured, and the patient has re-emerged in an improved version.

The holistic process theory thus implies that the patient must work with body, feeling, and mind at the same time. A practical solution is to let the patient draw on a team of therapists, some specialized in work on the body, others in words and feelings, and yet others in the mind and life philosophy. At the Quality of Life Research Center and Clinic in Copenhagen, the patient typically will see a body therapist, a psychotherapist, and a physician trained in the holistic-oriented approach, who also is in charge of referrals and the overall therapy. In our experience with this kind of work, a course of treatment to improve quality of life typically lasts about 6 months, consisting of 10 to 15 individual sessions at 2-week intervals. The treatment should be supplemented by reading relevant literature, perhaps a course in life philosophy and possibly participation in a "growth and development group" directed by a psychotherapist. In this group, patients join and support each other in the development process.

Regardless of their practical focus, the holistic treatment regime shares the approach of working with the patient rather than with the disease. The focus is on improving the quality of life. That is the reason why we often say "quality of life as medicine", when we explain the concept of consciousness-based medicine.

## THEORY OF COGNITIVE DISSONANCE

Let us take a closer look at what will happen when a person establishes the traumas that the holistic process theory seeks to eliminate. Let us draw on the classic sociopsychological theory of cognitive dissonance[26]. According to this theory, a person has a number of cognitions at any time, i.e., beliefs, attitudes, and perceptions. These cognitions may be more or less inconsistent. When there is a conflict between them, which Leon Festinger (1919–1990) from Stanford University calls cognitive dissonance, this is perceived as discomfort. Festinger viewed people as thinking individuals who need to have balance in their thoughts as well as their actions. This idea of balance is key to his theory of cognitive dissonance. Much research is still being conducted today in social psychology to answer some of the questions that cognitive dissonance has raised.

Let us look at an example: a small child begins by having the attitude "I am worth loving". But if the child feels constantly punished by its father, the child will acquire the experience "I see that I am not loved". Such dissonance is unpleasant and, according to Festinger, the child will attempt to change it to create consonance (harmony) between the cognitions. If the child cannot make the father stop the punishment so that the child achieves the cognition "I experience being loved", it is forced to change the cognition that it controls, i.e., the attitude "I am lovable". Consequently, the child will gradually change it into "I am unlovable". Now there is cognitive consonance between the child's two cognitions: "I experience being punished" and "That is because I am unlovable".

Festinger used his theory to explain why and how we change our own and others' attitudes and values (cognitions). It provides an excellent framework for our observations at the clinic of both traumatized children and adult patients reliving a traumatic childhood. However, we will demonstrate further below that patients often harbor numerous, mutually conflicting perspectives. Not until the traumatic material becomes conscious can the patient heal and become himself again. This healing can only take place once the gestalt — the painful "frozen now" — has moved from *the body,* where it is apparently stored and kept, and into the *emotional* dimension, where the old painful feelings should be contained and confronted, and further into *the mind* as a clear acknowledgment that can make us reassess our old choices of existential survival.

If we come to grief so early in life and make such self-destructive decisions as is often the case — I am unlovable, I am no good, I am hopeless, there is something wrong with me — we take vast amounts of vital energy from our living and bind this life force in unfinished gestalts. If we are to get well again and regain our vital energy, we have to melt the ice cubes of the past and in this way restore the exchange of information in the body. We must make the separate "parts" of ourselves, our denied sides, merge into our whole again. That is how we heal and regain our health and life force.

"The frozen now" is the essential element that binds our vital energy, and that is conventionally called a "gestalt" (we sometimes use the phrase holo-gestalt, when body, feeling and mind are involved at the same time). The gestalt begins as a pain that is unbearable, the next step is a life lie that relieves us from responsibility for the pain, and the third step is the parking of the entire gestalt in the part of the body that is able to contain it (typically a group of muscles or another organ structure).

## TOOLS

> "Please – tame me!" he said.
> "I want to, very much," the little prince replied. "What must I do, to tame you?" asked the little prince.
> "You must be very patient," replied the fox. "First you will sit down at a little distance from me – like that – in the grass. I shall look at you out of the corner of my eye, and you will say nothing. But you will sit a little closer to me, every day..." (Saint-Exupéry: The Little Prince[27])

According to Irvin D. Yalom, Emeritus Professor of Psychiatry at Stanford University School of Medicine[28], the good therapist must invent a unique treatment for each patient and the only thing that can really make a difference is attention, as Jiddu Krishnamurti (1895–1986)[29] pointed out throughout his life. Nevertheless, there appears to be some more concrete tools that the therapist can acquire and learn to master, in the interest of the patient.

When we meet a patient, typically the first thing we notice is how much closeness the patient allows between us. Some patients are good at closeness and intimacy; they have come far in their personal development and are very open and honest, both to themselves and to us. They trust in us and our good intentions as physicians and immediately accept our *attention, respect, care, acknowledgment, and acceptance*, which we consider the five fundamental therapeutic tools that any holistic doctor has to master to provide optimal holding, and on which every treatment should be based. The trusting patient often makes rapid progress if the therapist succeeds in combining these fundamental qualities in a smooth and unbiased manner, in the exact proportions needed by the patient, guided by the therapist's kindness towards — or perhaps even love of — the patient.

Other patients show little trust; they are emotionally distant and characterized by being equally unable to give and take. Attention hurts them, so we need to be very careful when we show them our attention and treat them. We view these patients as severely damaged. In such cases, the first therapeutic goal is to restore their trust by supporting them to feel, acknowledge, and let go of the trust-damaging traumas. The most important tools to achieve this goal are *touch, conversation, and sharing of life perspective.*

Touch comes at all levels of intensity, from the handshake to the embrace. Conversation comes in all degrees of intimacy, from the entirely impersonal to the most intimate conversation. Sharing of life perspective ranges from sharing of trivial, shallow views to generous sharing of our greatest moments and most significant experiences. People who cannot show trust can be very difficult to like and very difficult to help. The most damaged patients will not establish eye contact when they first visit the clinic; they are unable to see the world from any other viewpoint than the one in which they are stuck. We are not allowed to respect them because they cannot meet us at the line between us, but are either below or above that line. Nor are we allowed to touch them, for they cannot bear close contact with others. These patients may be normal in the psychiatric sense, but from an emotional view they are terrible, and from an existential view they are very ill. They have withdrawn from life completely and are stuck in a negative, mental position of defense.

> One patient is an example, a 50-year-old man, whom I (SV) perceived as totally impossible. All that I was allowed to do was to share his perspective, namely that he wanted to die. I could not talk him out of it in any way. I ended up giving him the only thing that I could, namely my meeting him concerning his death. Intuitively I felt that meeting him there was the only right thing to do, so we discussed suicide methods to find the exact method that would best suit him. Then, gradually I felt how his basic attitude changed: since I no longer tried to pull him from the grave, but accepted his independence and will to die and cooperated with him on his departure, part of him awoke. Ever so slowly he started struggling to live, rather than struggling to die. At the end of the session he declared that he no longer wanted to die.

We had but a single tool, one single straw to which the physician could cling as therapist, namely to join him unreservedly and without resistance where he was, in *his* mental perspective. The attitude and, in a way, generosity of the physician at this point apparently set him free. Half an hour later he was laughing at himself and his futile suicidal thoughts and the crisis had passed. The physician used his last and perhaps most important tool, namely to look at the situation from the patient's point of view, supported by the *intention* to help him.

Ideally, the holistic therapist is able to support the patient in all the processes concerned with feeling, acknowledging, and letting go. The goal of the therapy is to help the patient return to his or her natural state, where the patient surrenders to life and finds peace in life. The *care* of the physician helps the patient to be present in order to feel what is hidden away in the body. The *respect* of the physician enables the patient to establish his own well-defined space, which in turn enables the patient to understand himself and his life. The *attention* of the physician enables the patient to see himself and his life from many perspectives and to choose the very best and most affirmative views. Care, respect, and attention will not help the patient until he can accept them, and that is a question of trust. Trust enables the patient to accept the most nourishing support (called "holding"), which makes the deep and spiritual processes take their course.

Holding requires that the patient lets himself "be held", i.e., surrenders and lets the therapist take full control of the situation. That is the control held naturally by the parents before the fundamental breach of trust occurred. Usually, the patient would not let the parents or anyone else take control ever again. Healing takes place through surrender, where the patient once more lets go of all the ways he or she holds back: physical tension, emotional dissociation, and all the mental reservations. When the patient lets go, life returns. Healing will only be possible when the patient can accept being held, almost like a trusting infant. These resources — met with respect and love, being seen, touched — are the very resources that were missing in the original traumatic situation in life. In the successful cases, the ability to be close is recreated slowly, but steadily.

It is quite remarkable that one is able to provide parenthood with a delay of 30, 50, or 70 years and in that way make up for a terrible, traumatic loss of love and closeness in the past of the patient that has damaged the patient right up to the present. In therapy, the road to sufficient trust and profound healing is often long. Some of our patients have already been in therapy for many years before seeing us, without having found help in relation to their fundamental problems. The first 3 months are sometimes spent unlearning bad habits and misapprehensions that the patient has picked up during therapy or in other contexts.

Just how long that road is seems to be a question of the therapist's professional skill, emotional generosity, and intention. The therapist may be infinitely wise, but to no avail if he is unable to give. The ability to give must be sincere and loving. To be a good holistic therapist, one must make sure that one's own feelings are available for the encounter with the patient and that one is aware of one's own intentions. One must be able to withstand being reflected by the patient. One must master the classic concepts, such as transference and projection. As a person, the therapist should be attentive, respectful, and caring. Then we can begin practicing the three tools mentioned above that create trust and closeness. Let us take a closer look at them.

## 1. Touch

A caressing touch is the essence of care and as such one of the most natural and vital things for us as children. Most of us do not receive the care we need as children and are still deeply hurt. Therefore, when the therapist touches his patient, there is a risk that he may open some of the most painful wounds and expose the patient to more than he or she can handle. If we are not attentive and respectful, and attain the patient's complete acceptance, it may easily seem like an assault. On the other hand, there is no other way of healing the old wounds than to go back and relive the pain. Therefore, there is no alternative to touching the patient. The simplest and most natural thing is thus the hardest for us as therapists in the clinic. It is in the touching of our patient that we prove our worth as holistic physicians.

When supporting the patient to feel what lies hidden in the body — as being taught by Marion Rosen who developed the Rosen method[30] — the holistic therapist should place his hands on the patient and support the tense, blocked, and perhaps diseased area of the body. The purpose of the touch is to meet the patient soul to soul through the two bodies. In order for the touch to establish contact with repressed matter, the therapist's genuine intention with the touch should be to encounter the patient's self, not just the patient's body. Therefore, the therapist has to recognize qualities in himself, such as curiosity towards the other person, pleasure in the contact or wanting to touch, all qualities which are considered unwanted and unwelcome in general medical practice. Marion Rosen[30]: "When you touch the client, the client also touches you". That is true, which for a while can cause some difficulties because it means that a therapeutic touch performed correctly in the holistic context leads to uncompromising and quite provocative closeness. When the patient, slowly and step by step, becomes present in a sick and blocked area, the repressed feelings and problems, which are often placed there in early childhood, will slowly rise to the surface of consciousness. The patient becomes present and meets the therapist and the gestalt that was hidden in the tissue resurfaces with all its negative feelings. Mostly, this process is gradual and quiet, but sometimes it is explosive. And then the trick is not to comfort the patient.

We begin the physical touch of the patient in a fairly neutral place, on the hands or knees; on the couch we proceed to touch the head, chest, and abdomen, corresponding to the third eye, the heart/solar plexus and the Hara center. When we touch a patient on the forehead and a handbreadth below the navel, this double touch can lead to deep contact, which triggers the holistic healing process. Two dormant intelligences, intuition in the body and the analytical intelligence in the mind, can be activated in this way, and applying these talents may really speed up the patient's process.

Touch combined with conversation can change the patient's well being radically over a short time. It is always surprising for the patient when an opening appears and feelings return. Not always pleasant, of course, as the patient finds the feelings exactly the way they were left — often in unbearable pain. The patient may suddenly begin to laugh and then to cry bitterly on the couch in a way that the patient has not cried for half his life. The "feel therapy" is the port of entry. Without it, there is no hope of therapeutic progress in the context of consciousness-based medicine.

## 2. Conversation

The holistic conversation has the same purpose as holistic touch: soul-to-soul contact, this time through our two minds. Conversation has it all: body, feelings, and thought. A voice contains so much body, so much feeling, with so much to tell about the person it belongs to. And the choice of words tells its own story, the tone of voice and finally the sentences and the intention behind them that carry them forth. It is tempting to believe that conversation embraces all of it. What else is needed when we meet, but to speak together sincerely, openly, honestly, and intimately?

In the same way that touch becomes extremely difficult due to old neglect, the conversation which is supposed to be so natural becomes so difficult because of all the abuse, verbal assaults, and mental defeats that we have suffered in our lives. Conversation is therefore also a door to awareness, but many feelings and events are so painful or so well repressed that they are actually unreachable through conversation and memory. As with touch, important questions relate to conversation. What happens when two people are good at talking to each other and what is it about conversation that really redeems and develops the patient? How do we help a person towards consciousness and clarification?

In our experience, the conversation does not take on real value until the patient begins to express himself. It seems that every person contains a very large, inner truth and only when it is verbalized can the person live and apply that truth. The conversation should support the patient to find his own unique and original expression, which is verbal, emotional, and spiritual all at once. We really mean something; we are not vacillating or indifferent. Deep down, we represent something. Finding this content in life, verbalizing what we feel and think so that our mind become clear, that is where conversation supports the patient. In a way, each fruitful conversation is like a vortex, pulling the meaning and content towards its center and down towards the depth of the soul. And not until the basin is emptied and the vortex has become completely still and is completely centered in it own center, is the process over. The person has become aware, emotionally focused, and conscious. The patient has “opened up his heart” as the feelings returned, and mind and body reach each other once again. The opening of the heart and the recuperation of feelings reopen the door to the depth of existence, the wholeness of man, or the soul.

## 3. Setting Perspectives

Generally, we may not notice that our attention changes its nature in relation to what we do and the place within us from which we work, but this is a very important point in the holistic clinic. Touch, for instance, works best when the physician is centered in the abdomen and pelvis, in the Hara center, which is our center of being and physical desire. Conversation is most constructive and natural with the center in the heart and solar plexus, since the heart creates connection and the solar plexus creates clarity and definition.

When we become centered in the center of the mind, traditionally called the third eye, we gain access to the quality we call acuity. It is a frightening quality that relates to making conscious choices. What we can choose in a state of acuity is our philosophical position, our perspective. It appears that over time most people move away from a perspective close to life, often towards a very strange perspective distant from life.

When we, as holistic physicians, are to help our patients, perhaps our strongest tool is the awareness of life perspective and the invitation to a shift in perspective. Once we have gained the patient’s trust, we can make a journey together, exploring a number of alternative life perspectives and their consequences for our lives. It often comes as a shock to discover the decisive influence of our personal life perspective on our quality of life and well being. If we succeed in helping the patient return to his or her natural life perspective, which is in full harmony with the inner life, this patient will often get well. As the patient lets go of all mental difficulties and firm views, he or she will return to the natural life perspective that expressed the patient’s inner truth. At last the patient can experience the correlation between the inner and outer life, which was disturbed temporarily by the old traumas. The inner conflicts and contrasts dissolve, and the patient can experience happiness, perhaps for the first time. When we succeed in helping the patient become focused in his or her own mind, we say that the patient has become conscious. From that moment on, the patient is in control of his or her own destiny. Choosing a life perspective that is in harmony with one’s inner life means *taking responsibility for life*.

Let us look at an example of this. The patient in the following case presented with a terribly disfiguring scar in the lower part of the face. She wanted to die because she looked so ugly that nobody could love her, not even her own parents. The physician (SV) first made her choose to live by setting a dramatic perspective where she was made aware of the choice between life and death, and she chose life. Subsequently, he applied touch, with her permission, first resting his hands on her old disfiguring scar and then massaging the scarred tissue. She starts crying hysterically as she spontaneously goes back to the time when she suffered burns. The re-experience is so intense that her lips turn blue again and she gasps desperately for air as if the flames prevent her from breathing here and now.

> **Female, aged 20 years – healing scars on face after severe burns.** First session. She had a large scar on her face after suffering burns at the age of 3. On the couch, therapy centers on spontaneous regression to the episode with massaging of the scar, which healed nicely in terms of energy. She cried and relived a lot of suffering, which was processed. Before this we talked about choosing a life of suffering or a peaceful death. She disclaimed responsibility based on karma theory [it is not her fault that things are the way they are, it is because of her bad karma, i.e., the consequences of harmful actions in

> previous lives that pursue her], which we discussed. Another appointment in 2 weeks. EXERCISE: Write a complete list of all your problems in life. Write half a page on each problem.

The physician touched her and she felt. The healing process was underway. The patient must then be supported in acknowledging and she has to verbalize the difficult feelings that emerged. The physician then joined her in her feelings. It was important that the physician accommodate the patient with all her feelings, allowed her to express herself and her feelings in the situation, even if she was very childish, very aggrieved, and very hurt. All these feelings have nothing to do with the physician, they are things of the past, which the patient really needed to talk about and express. This took place during the second session with the patient.

> Second session. She has done her homework — about 10 pages about her problems — she will type it out and improve it for next appointment. Her father abused her sexually when she was 16 years old. “Perhaps before that, too?” the patient asked. Has a boyfriend, but right now neither of them knows whether they should keep seeing each other. She is very dependent on the security and closeness that she gets with him. She feels very sorry for herself. Her wicked mother has ruined her life. EXERCISE: Write an essay on your self-pity. Also write a little about your conceitedness. Next time: We continue to work on the scar. Have a portrait taken that clearly shows your scars.

In helping the patient to acquire a more down-to-earth and constructive life philosophy, we have to join her in her perception of reality. Again, we can meet soul to soul, this time through the mind, and together examine whether the patient’s present life perspective is the view of life that she basically has and wants to have. Massive self-expression concerning life perception, perhaps in the form of a written biography, promotes this process considerably. The therapist must be able to accommodate even the most sinister view of life and existence to help the patient acquire a more down-to-earth life perspective. In this session, preparations are made for the major shift in perspective, which the patient needs in order to regain her enjoyment of life.

> Third session. She has done her homework — about ten A4 pages, partly about self-pity. The patient’s name used to be P, but she went to a numerologist and then changed her name to the present one. That way she felt that she got away from her old life. It felt good. “Have barely seen my parent for a couple of years – they sort of destroyed me,” she says. She cries and refuses to take responsibility for her past. EXERCISE: Write from the present and go back through all the major events that have evoked feelings in you: what happened, what did you feel, what happened, what did you feel, etc. – and what did you decide in each situation. May take up to 100 pages. One hour a day. Another appointment in 2 weeks.

To help the patient experience a real breakthrough to herself, she now has to begin sorting out everything that keeps her from being herself. It is a vast job, but it can be done. Now our job is to motivate her to make a persistent and substantial effort, perhaps for a year or so, until she finally and inevitably breaks through to her real self. A patient who chooses the soul’s perspective often experiences a more profound meaning of life, joy in being alive, and fundamental peace and being. It feels like coming home, like knowing oneself again, like being back in control. Like this statement from one of our patients: “I haven’t felt like this before. I have always been insecure. People have always led me by the nose. Now it is completely different, and now *I* am back in charge. And I am happy.”

This experience of things falling into place in life triggers an inner revolution, which frees the self-healing powers of the body and mind. Personal development is a life-long process, but the road to health need not be as long. And in the long term, we also develop good quality of life and good functional capacity — at work, socially, or sexually. Therefore, we believe holistic medicine is a key to the good life in a broad sense and also a key to help people become useful to and coherent with those around them and society. Holistic medicine is thus also a sustainable project of public utility, which optimizes the value of the individual in relation to its fellow human beings, society, and the ecosystem.

It should be emphasized that the starting point of consciousness-based medicine also includes the individual's physical and mental appearance, first and foremost the body, which can be regarded as a direct manifestation of the patient's consciousness. In our view, the body and feelings contain just as much consciousness as the head and mind. That is because our consciousness cannot be narrowed down to the mind and therefore does not reside in the head, but indeed in the whole that embraces all our parts.

It is an interesting fact that what patients need to heal spontaneously is the combination of attention, respect, and care. And it is an interesting fact that this very combination cannot be given without profound and genuine love of the other person. What may appear to be technique and scientific knowledge in the eyes of the young therapist, gradually — as experience and wisdom grow — increasingly resembles genuine love and kindness towards one another. Consciousness-based medicine thus moves towards what we call social utopia. We consider the holistic process the first step towards consciousness-based medicine.

## DISCUSSION

Biomedicine focuses on the biochemistry of the body, while consciousness-based medicine (holistic medicine) focuses on the individual's experience and conscious whole (Greek: *holos*, whole). Biomedicine perceives diseases as mechanical errors at the micro level, while consciousness-based medicine perceives diseases as disturbances in attitudes, perceptions, and experiences at the macro level — in the organism as a whole. Thus, consciousness-based medicine is based on the whole individual, while biomedicine is based on its smallest parts, the molecules.

These two completely different points of departure make the two forms of medicine very different, they represent two different mind sets and two different frames of reference or medical paradigms. Some of the differences may be listed as follows:

| **Biomedicine** | **Consciousness-Based Medicine (Holistic Medicine)** |
|---|---|
| The physician helps you | The physician supports you in helping yourself |
| The physician is responsible for how you are doing | You are responsible for how you are doing |
| The physician treats you with medicine and surgery | You develop by feeling, understanding, and letting go of negative perceptions |
| Based on body and compliance | Based on consciousness and learning |
| Disease controls the individual | Health and quality of life are created through personal development |
| Peace and quiet to recover | Inspiration to rediscover hidden resources |
| Focus on lifestyle and physical factors | Focus on life philosophy, wholeness or " soul", responsibility, love, respect, and care |
| Your genes determine how beautiful, good, and true you are | Your degree of inner consonance determines how beautiful, good, and true you are |

The greatest and most conspicuous difference between biomedicine and consciousness-based medicine concerns the perception of resources. According to biomedicine, you need support in the form of chemical substances when you are ill or weak, while according to consciousness-based medicine you need to mobilize your hidden resources. This does not imply that a holistic physician will not prescribe penicillin to treat pneumonia, or that the biomedically oriented physician will not talk to his patient. But it does imply a totally different perception of the resources required to help the patient get better and function better.

Another highly striking difference between biomedicine and consciousness-based medicine is the relationship with quality of life. To biomedicine, good quality of life is a result of health, meaning that the patient's quality of life will improve when the doctor treats the body or mind with medicine. To the holistic doctor, improvement of the quality of life is the very key to mobilizing the hidden resources. To the biomedical doctor, increased quality of life is a result of improved health, while to the consciousness-oriented doctor improved health is a result of increased quality of life. According to consciousness-based medicine, the patient acquires quality of life when he or she takes responsibility for his or her life. According to biomedicine, the patient acquires quality of life when the doctor takes responsibility for the patient. Hence, the perception of the patient's responsibility for his or her own life and illness is another important difference between biomedicine and consciousness-based medicine.

Therefore biomedicine may be defined simply as medicine based on a biochemical perception of man: we are chemical machines. Similarly, consciousness-based medicine is founded on the consciousness of man: we are conscious beings who choose our own lives and thereby to a great extent create our own lives. The biomedical perception of reality is that everything consists of atoms and that experiences and consciousness are kind of by-products of the chemical processes in the brain. The brain chemistry makes all decisions, consciousness actually lags far behind, but merely imagines that it matters. Consciousness-based medicine considers consciousness to be just as real as, but not more real than, substance. Consciousness is a real phenomenon, an element, if you will, in the same way as atoms. And our consciousness has great influence. Through all our minor and major conscious choices, consciousness is the primary cause of our present lives.

In this paper we have compared biomedicine and consciousness-based holistic medicine. It is very important to stress that we imagine the excellent physician to use both toolboxes and more[31] in his treatment of his patients; we actually want the physician to be *multiparadigmatic*[32]. Often both biomedicine and holistic medicine must be taken into use to cure a patient; mostly the acute problems can be solved using biomedicine, while chronic health problems need a holistic approach[33,34,35,36,37].

Using holistic medicine and therapeutic touch — being very intimate with and very close to the soul of the patient, emotions, and body — is only possible with an ethical consciousness on the part of the physician[31,37]. We strongly believe that the results of a holistic physician never will be better than his ethical standard.

There are many great theories and philosophies in favor of a holistic approach to human health, like the works of Maslow[38], Antonovsky[39,40], Frankl[41], and Jung[42], but what is a thousand times more important for medicine than the opinions and perspectives of wise old men is your own understanding in the daily clinical practice of what it takes for you as the physician to cure your patient and make him or her well again.

## CONCLUSION

One of the great advantages of consciousness-based medicine is that, in an abstract sense, the physician should always do the same thing regardless of what is wrong with the patient, namely support the patient in becoming more conscious, more whole, and more himself. Biomedicine often requires advanced technological assessment programs and completely accurate diagnoses prior to implementing a successful treatment. In the field of consciousness-based medicine, it is far more important — as Hippocrates (460–400 BCE) already taught us — which person has a disease than which disease a person suffers from. The holistic medicine is basically, as it was in the days of Hippocrates, about the recovery of the human character, serving the realization of our purpose of life.

## ACKNOWLEDGMENTS

This study was supported by grants from IMK Almene Fond. The quality of life research was approved by the Copenhagen Scientific Ethical Committee under number (KF) V.100.2123/91.

## BIOSKETCHES

**Søren Ventegodt, MD,** is the Director of the Quality of Life Research Center in Copenhagen, Denmark. He is also responsible for a Research Clinic for Holistic Medicine in Copenhagen and is a popular speaker throughout Scandinavia. He has published numerous scientific or popular articles and a number of books on holistic medicine, quality of life, and quality of working life. His most important scientific contributions are the comprehensive SEQOL questionnaire, the very short QoL5 questionnaire, the integrated QOL theory, the holistic process theory, the life mission theory, and the Danish Quality of Life Research Survey, 1991–94 in cooperation with the University Hospital of Copenhagen and the late pediatric professor Bengt Zachau-Christiansen. E-mail: ventegodt@livskvalitet.org. Website: http://www.livskvalitet.org

**Mohammed Morad, MD**, is Specialist in Family Medicine, Lecturer in Family Medicine at the National Institute of Child Health and Human Development, Division of Community Health, Ben Gurion University of the Negev and the Medical Director of a large area clinic in the city of Beer-Sheva. He has publications on Bedouin health, health aspects, spiritual health, and aging in persons with intellectual disability, and is a presenter on topics such as health policy and services for the disadvantaged at national and international conferences. E-mail: morad62@barak-online.net

**Niels Jørgen Andersen, MSc,** Professor, Department of Innovation and Economic Organization, Norwegian School of Management. This department conducts research and provides teaching in central topics related to innovation, business development, management of global companies, business history, and economic organization. Research activities within the Department are related to four core subjects within the discipline: business history, cooperative organizations, business development and entrepreneurship, and finally studies of industries with a special focus on the electricity industry. He is also the dynamic chairman of the nonprofit organization Stiftelsen Holistisk Medisin Scandinavia, that aims to support the scientific development, research, and documentation of complementary and holistic medicine in Scandinavia. E-mail: niels.j.andersen@bi.no. Website: http://www.bi.no/users/fgl93013

**Joav Merrick, MD, DMSc,** is Professor of Child Health and Human Development affiliated with the Zusman Child Development Center, Division of Pediatrics and Community Health at the Ben Gurion University, Beer-Sheva, Israel; the Medical Director of the Division for Mental Retardation, Ministry of Social Affairs, Jerusalem; and the Founder and Director of the National Institute of Child Health and Human Development. He has numerous publications in the field of child and human development, rehabilitation, intellectual disability, disability, health, welfare, abuse, advocacy, quality of life, and prevention. Dr. Merrick received the Peter Sabroe Child Award for outstanding work on behalf of Danish Children in 1985 and the International LEGO-Prize ("The Children's Nobel Prize") for an extraordinary contribution towards improvement in child welfare and well being in 1987. E-mail: jmerrick@internet-zahav.net. Website: www.nichd-israel.com
